# Early caffeine therapy decreases bronchopulmonary dysplasia but might increase mortality in preterm infants? a systematic review and meta-analysis

**DOI:** 10.3389/fped.2025.1528054

**Published:** 2025-02-21

**Authors:** Juan Ma, Long Chen, Kaihong Mu, Shifang Tang, Yuan Shi

**Affiliations:** ^1^ Department of Neonatology Children's Hospital of Chongqing Medical University, National Clinical Research Center for Child Health and Disorders, Ministry of Education Key Laboratory of Child Development and Disorders, Chongqing Key Laboratory of Pediatric Metabolism and Inflammatory Diseases, Chongqing, China; ^2^ Department of Pediatrics, SongShan General Hospital, Chongqing, China; ^3^ Department of Neonatology, Women and Children’s Hospital of Chongging Medical University (Chongging Health Center for Women and Children), Chongqing, China

**Keywords:** caffeine, bronchopulmonary dysplasia, mortality, meta-analysis, BPD

## Abstract

**Objectives:**

To assess the effectiveness of early vs. late caffeine therapy for bronchopulmonary dysplasia (BPD) in infants.

**Methods:**

PubMed, Embase, Web of Science, and Cochrane databases were searched up to October 2024. Studies comparing early and late caffeine therapy for BPD in infants were included. The primary outcomes were the incidence of BPD, severe BPD, and mortality.

**Results:**

Eleven studies (1 RCT and 10 cohorts) with 64,749 patients (34,175 early and 30,574 late) were included. Meta-analysis revealed a significantly lower incidence of BPD (OR: 0.67; 95% CI: 0.56, 0.79; *P* < 0.00001) but higher mortality (OR: 1.20; 95% CI: 1.12, 1.29; *P* < 0.00001) in the early group. Subgroup analysis showed a significant difference in BPD incidence in retrospective studies (OR: 0.57; 95% CI: 0.44, 0.74; *P* < 0.0001), but not in prospective studies (OR: 0.84; 95% CI: 0.44, 1.61; *P* = 0.61). No significant difference was observed in severe BPD incidence (OR: 0.89; 95% CI: 0.34, 2.35; *P* = 0.81).

**Conclusions:**

Early caffeine therapy may reduce BPD incidence but increase mortality risk in infants. More large-scale, prospective studies are needed to further evaluate the efficacy of early vs. late caffeine therapy for BPD.

**Systematic Review Registration:**

https://www.crd.york.ac.uk/prospero/display_record.php?RecordID=474351, PROSPERO (CRD42023474351).

## 1 Introduction

Bronchopulmonary dysplasia (BPD) is a common respiratory complication in premature infants, particularly those with ultra-low and very low birth weights. Its incidence is inversely correlated with birth weight and gestational age (1). BPD typically arises from deviations in normal lung development, influenced by preterm birth itself as well as various potential interfering factors, including genetic factors (2). Pathological changes associated with BPD include alveolar simplification, reduced alveoli count, abnormal capillary development, and aberrant growth of vascular tissue (3). Preterm infants with BPD often exhibit symptoms such as wheezing, decreased gas exchange capacity and mobility ability, along with diminished quality of life. Mild to moderate respiratory obstruction and airway collapse may occur in children aged 1–2 years. Significant respiratory obstruction and airway hyperresponsiveness can be observed in school-aged children who continue to grow; during adolescence follow-up visits most patients develop chronic obstructive pulmonary disease similar to that seen in adults. After puberty onset the majority of children experience abnormal lung function and emphysema (4–6).

The drug prevention and treatment of BPD involves the administration of glucocorticoids, vitamin A, macrocyclic vinegar, etc. Among these options, glucocorticoids are the most commonly utilized in clinical practice (7). However, due to recent reports of adverse reactions and potential long-term neurodevelopmental outcomes associated with their use, the clinical application of glucocorticoids remains controversial (8). Caffeine has been studied as an adenosine receptor inhibitor since 1977 for its role in premature apnea. It has been found that caffeine can reduce the duration of respiratory support treatment, improve lung function, and to some extent decrease the incidence of BPD later on (9). Nevertheless, there is no consensus regarding the optimal timing for administering caffeine treatment for BPD. Studies have demonstrated that caffeine not only helps prevent neonatal apnea but also shows positive effects in preventing and treating both BPD and patent ductus arteriosus (PDA). Early initiation of caffeine therapy (within three days after birth) significantly reduces the occurrence of BPD (*P* < 0.01) as well as decreases the need for PDA medication or surgical intervention (*P* = 0.01) (10–13). However, it should be noted that several studies have indicated that early use of caffeine does not appear to significantly reduce the incidence of BPD in preterm infants (14, 15). Furthermore, two large cohort studies have suggested a potential increase in all-cause mortality among preterm infants who received early caffeine therapy; however, this finding may be influenced by confounding factors (10, 13).

Therefore, the impact of caffeine treatment timing on mortality and the incidence of BPD in preterm infants remains inconclusive. This study aim at conducting a meta-analysis incorporating the latest and most comprehensive clinical data to establish evidence-based findings regarding the association between caffeine administration timing and BPD incidence and mortality.

## 2 Methods

### 2.1 Literature search

The present meta-analytic investigation adhered to the PRISMA (Preferred Reporting Items for Systematic Reviews and Meta-Analyses) 2020 guidelines (16) and was prospectively registered in PROSPERO (CRD42023474351). PubMed, Embase, Web of Science, and Cochrane databases use to search literature up to October 2024, to identify studies that evaluated the effectiveness of initiating caffeine therapy at an early stage compared to a later stage for the management of BPD in infants. Searching terms: “Caffeine”, “Methylxanthine”, “Xanthine” and “Bronchopulmonary Dysplasia”. The detailed search strategies in PubMed are as follows: ((((“Caffeine”[Mesh]) OR ((((((((((((No Doz) OR (Caffedrine)) OR (Coffeinum N)) OR (Coffeinum Purrum)) OR (Dexitac)) OR (Durvitan)) OR (Percoffedrinol N)) OR (Vivarin)) OR (Percutaféine)) OR (Quick-Pep)) OR (QuickPep)) OR (Quick Pep))) OR (“methylxanthine” [Supplementary Concept])) OR (“Xanthine”[Mesh])) AND ((“Bronchopulmonary Dysplasia”[Mesh]) OR (BPD)). The detailed information were presented in Supplementary Table S1. Two investigators screened all included RCTs independently, and via discussion to solve the differences.

### 2.2 Inclusion and exclusion criteria

Inclusion criteria: (1) RCTs, cohort, case-control study; (2) studies in infants; (3) comparison of early (no later than 3 days after birth) vs. late caffeine (no earlier than 1 day after birth) therapy efficacy; (4) evaluation of at least one outcome; (5) sufficient data to analyze odds ratio (OR), weighted mean difference (WMD), or standard mean difference (SMD). Excluded were protocols, unpublished studies, non-original works (letters, comments, abstracts, corrections, and replies), studies lacking sufficient data, reviews, case reports, and studies with low quality. In this meta-analysis, BPD is defined as preterm infants less than 32 weeks of gestational age who require supplemental oxygen for at least 28 days after birth, with mild BPD as oxygen requirement at diagnosis (breathing ambient air), moderate BPD as less than 30% oxygen, and severe BPD as more than 30% oxygen (17, 18).

### 2.3 Data abstraction

Two authors extracted data independently, and third author solve disagreement. The following information was collected: first author, publication year, research period, study region, design, population, intervention (early caffeine), control (late caffeine), sample size, gestational age, gender, birth weight, 5-minute Apgar score, incidence of bronchopulmonary dysplasia (BPD), incidence of severe BPD, and mortality. Continuous data presented as median and range or median and interquartile range (IQR) were recalculated to mean ± standard deviation (SD) using methods by Wan et al. and Luo et al. (19, 20).

### 2.4 Quality evaluation

The quality of RCTs was evaluated according to the Cochrane Handbook for Systematic Reviews of Interventions 5.1.0, covering seven domains: random sequence generation, allocation concealment, participant and personnel blinding, outcome assessor blinding, incomplete outcome data, selective outcome reporting, and other bias sources (21). Each domain was classified as low-risk, high-risk, or unclear-risk, with more “low-risk” ratings indicating higher quality. The Newcastle-Ottawa Scale (NOS) was used to assess cohort study quality (22), with scores of 7–9 points considered high quality (23). Two authors independently assessed the quality, resolving disagreements through discussion and consensus.

### 2.5 Statistical analysis

Meta-analysis was conducted using Review Manager 5.4.1 software. For continuous data, weighted mean differences (WMDs) or standardized mean differences (SMDs) were used, and odds ratios (ORs) were applied for dichotomous data. Each measure was reported with 95% confidence intervals (CIs). Heterogeneity was assessed using the chi-squared (χ^2^) test (Cochran's *Q*) and the inconsistency index (I^2^) (24). Significant heterogeneity was defined as a χ^2^ *P*-value <0.1 or I^2^ > 50%. In the presence of significant heterogeneity, the random-effects model was employed; otherwise, fixed-effects model used. Subgroup analyses were conducted for outcomes with three or more studies to evaluate potential confounding factors and the source of heterogeneity when data were available. Sensitivity analyses with one-by-one elimination method were conducted to assess the impact of each study on the overall WMD, SMD, or OR and the source of heterogeneity for results involving more than two studies. Publication bias was assessed by generating funnel plots with Review Manager 5.4.1 and performing Egger's regression tests (25) using Stata 15.1 (Stata Corp, College Station, Texas, USA). A *P*-value <0.05 indicated significant publication bias.

## 3 Results

### 3.1 Literature retrieval, study characteristics, and baseline

**Figure 1:**
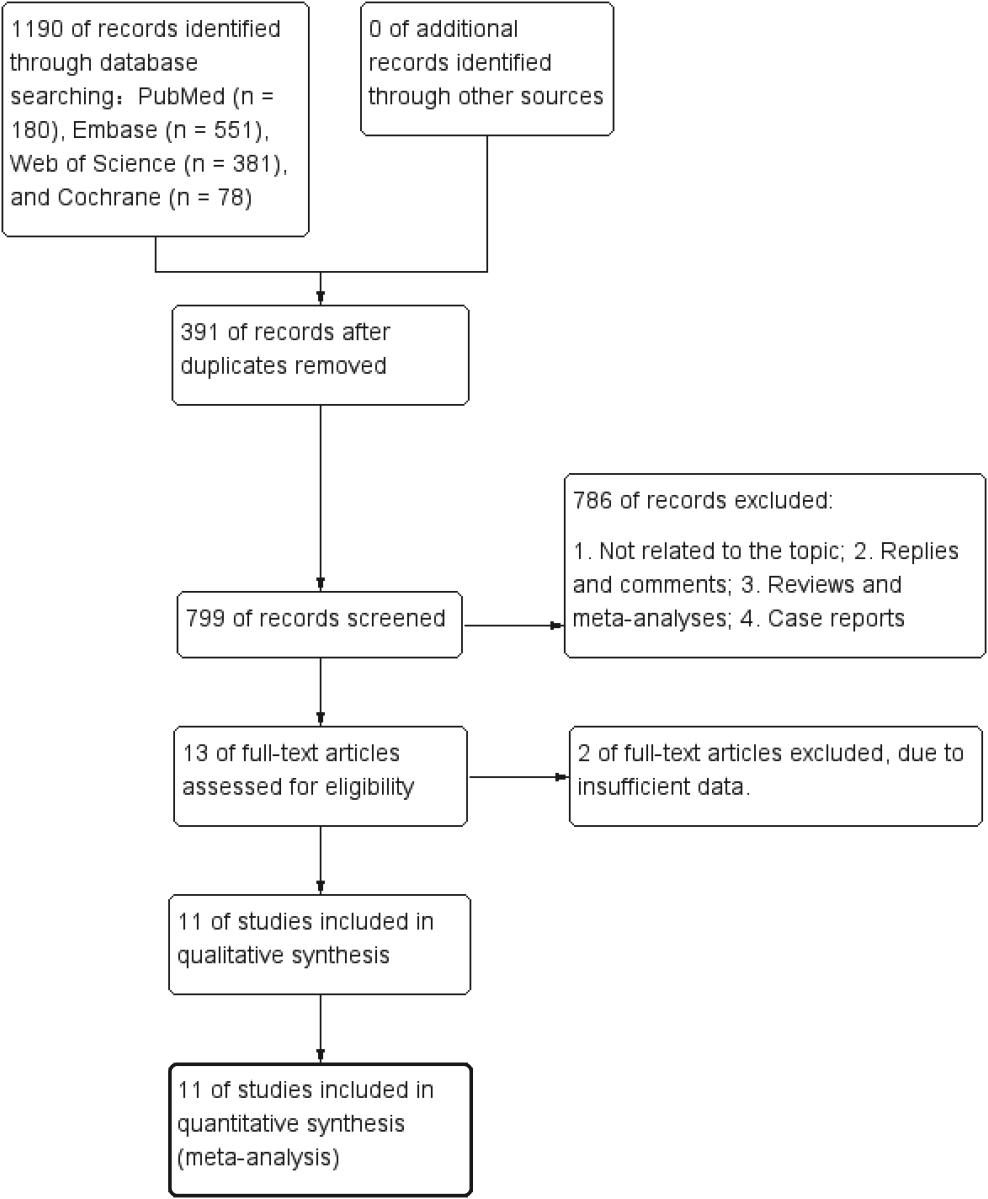
Flowchart of the systematic search and selection process.

The flowchart of the literature screening process was shown in Figure 1. 1190 studies were identified in PubMed (*n* = 180), Embase (*n* = 551), Web of Science (*n* = 381), and Cochrane (*n* = 78). After removing duplicates, 799 titles and abstracts remained. Ultimately, 11 studies (1 RCT and 10 cohort studies) involving 64,749 patients (34,175 in the early group and 30,574 in the late group) were included (1, 10–15, 26–29). The characteristics of each eligible study are presented in Table 1. Quality evaluation details for included RCTs are shown in Supplementary Figure S1 and Table S2 presents the quality evaluation for included cohort studies. Birth weight was similar between the two groups (SMD: 0.23; 95% CI: −0.001, 0.46; *P* = 0.05), as were gender (male) (OR: 1.00; 95% CI: 0.96, 1.04; *P* = 0.96), gestational age (WMD: 0.29; 95% CI: −0.09, 0.68; *P* = 0.13), and 5-minute Apgar score (WMD: 0.07; 95% CI: −0.18, 0.32; *P* = 0.60) (Table 2).

**Table 1:** Baseline characteristics of include studies and methodological assessment.

Authors	Study period	Country	Study design	Patients (*n*)	Population	Early caffeine	Late caffeine	Dose of caffeine	Quality score
				Early/late					
Borszewska-Kornacka (25)	2014–2015	Poland	Prospective cohort	143/143	Infants ≤32 weeks’ gestation with RDS	Initial dose on the 1st day of life	Initial dose on day 2 + of life	NA	8
Dobson (10)	1997–2010	USA	Prospective cohort	14,535/14,535	Very low birth weight (VLBW) infants	Initial dose before 3 days of life	Initial dose at or after 3 days of life	NA	7
Elmowafi (14)	2016–2018	Egypt	RCT	90/91	Infants ≤32 weeks’ gestation	In the first 72 h of life	Only if apnea exists or infant requires mechanical ventilation	Caffeine citrate was given as a loading dose of 20 mg/kg/day and a maintenance dose of 10 mg/kg/day.	–
Hand (15)	2010–2014	USA	Retrospective cohort	85/65	Preterm neonates with gestational age ≤29 weeks	Initial dose before 3 days of life	Initial dose at or after 3 days of life	NA	8
Lodha (26)	2010–2012	Canada	Retrospective cohort	3,806/1,295	Preterm neonates who were born at less than 31 weeks’ gestational age	Within the first 2 days after birth	On or after the third day following birth	The usual practice in most of units is to administer a loading dose of 10 mg/kg of caffeine base, with a daily maintenance dose of 2.5–5 mg/kg initiated 24 h after the loading dose.	6
Patel (11)	2008–2010	USA	Retrospective cohort	83/57	Neonates ≤1,250 g at birth	Initiation <3 days of life	Initial dose at or after 3 days of life	NA	7
Shenk (12)	2008–2014	USA	Retrospective cohort	80/58	Extremely low birth weight neonates	Days of life 0–2	Day of life 3 or greater	The secondary objective compared a larger initial caffeine maintenance dose (10 mg/kg day) to a smaller maintenance dose (5 mg/kg/day). All patients received a caffeine loading dose of 20 mg kg.	7
Szatkowski (13)	2012–2020	UK	Retrospective cohort	13,045/13,045	Infants ≤32 weeks’ gestation	On the day of birth or the day after	On or after day 3	NA	6
Taha (27)	2006–2011	USA	Retrospective cohort	1,986/965	Infants less than or equal to 1,250 g at birth	0–2 days of life	3–10 days of life	NA	6
Ye (1)	2017–2019	China	Retrospective cohort	227/225	Preterm infants with gestational ages less than 37 weeks	Initiating within 48 h after birth	Initiating over 48 h after birth	All preterm infants received a loading dose of caffeine citrate (20 mg/kg) through intravenous drip for 30 min, followed by a maintenance dose of 5–10 mg/kg daily, which started 24 h after the loading dose.	6
Yun (28)	2016–2018	Malaysia	Retrospective cohort	95/95	Preterm infants born at less than 37 weeks gestational age	Initiation within 2 days of life	Initial dose at or after 3 days of life	The early caffeine therapy group, caffeine was prescribed at 5 mg/kg, compared to 10 mg/kg in the late caffeine therapy group.	6

**Table 2:** Demographics and clinical characteristics of included studies.

Outcomes	Studies	No. of patients	WMD or SMD	95% CI	*p*-value	Heterogeneity			
		Early/late				Chi^2^	df	*p*-value	*I*^2^ (%)
Birth weight	7	4,382/1,804	0.23	[−0.001, 0.46]	0.05	42.16	6	<0.00001	86
Gender (male)	10	21,130/17,529	1.00	[0.96, 1.04]	0.96	10.15	9	0.34	11
Gestational age	8	6,368/2,769	0.29	[−0.09, 0.68]	0.13	62.34	7	<0.00001	89
Apgar at 5 min	8	20,820/17,247	0.07	[−0.18, 0.32]	0.60	130.73	7	<0.00001	95

### 3.2 Incidence of BPD

**Figure 2:**
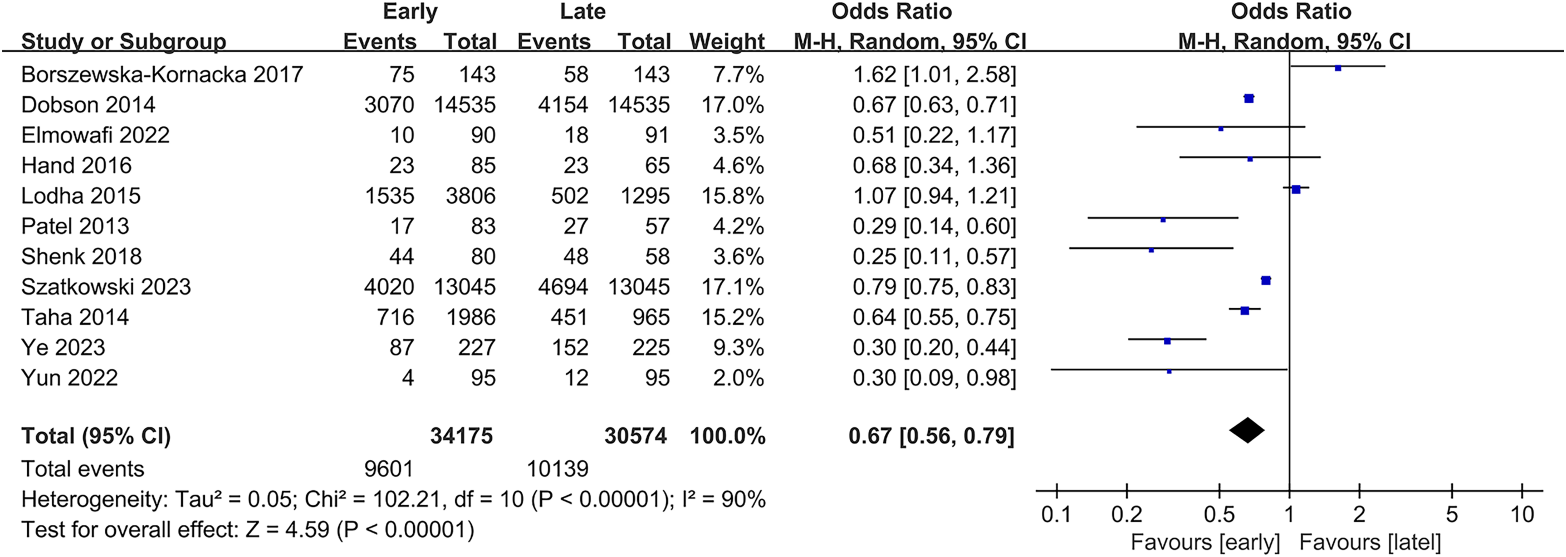
Forest plots of the incidence of BPD.

The incidence of bronchopulmonary dysplasia (BPD) was synthesized from 11 studies, involving 64,749 patients (34,175 in the early group vs. 30,574 in the late group) (1, 10–15, 26–29). Meta-analysis showed a significantly lower incidence of BPD in the early group (OR: 0.67; 95% CI: 0.56, 0.79; *P* < 0.00001) with considerable heterogeneity (I^2^ = 90%, *P* < 0.00001) (Figure 2).

Subgroup analysis revealed a significant difference in retrospective studies (OR: 0.57; 95% CI: 0.44, 0.74; *P* < 0.0001) (10, 14, 26), whereas significance disappeared in prospective studies (OR: 0.84; 95% CI: 0.44, 1.61; *P* = 0.61) (Supplementary Figure S2) (1, 11–13, 15, 27–29).

### 3.3 Incidence of mild and moderate BPD

**Figure 3:**
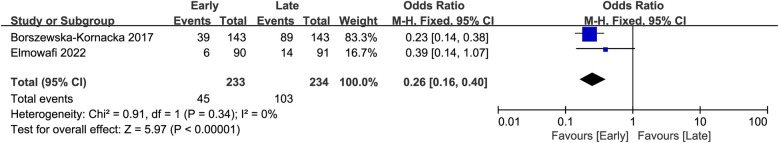
Forest plots of the incidence of mild and moderate BPD.

The incidence of mild and moderate BPD was synthesized from 2 studies involving 467 patients (233 in the early group vs. 234 in the late group) (14, 26). Meta-analysis showed a significantly lower incidence of mild and moderate BPD in the early group (OR: 0.26; 95% CI: 0.16, 0.40; *P* < 0.00001) without significant heterogeneity (I^2^ = 0%, *P* = 0.34) (Figure 3).

### 3.4 Incidence of severe BPD

**Figure 4:**
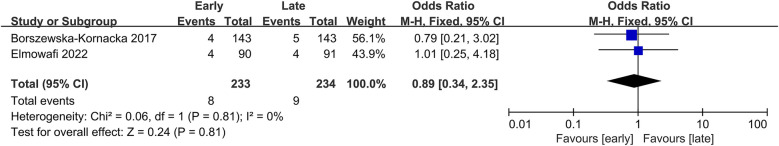
Forest plots of the incidence of severe BPD.

The incidence of severe BPD was synthesized from 2 studies involving 467 patients (233 in the early group vs. 234 in the late group) (14, 26). No significant difference in severe BPD incidence (OR: 0.89; 95% CI: 0.34, 2.35; *P* = 0.81) (Figure 4).

### 3.5 Mortality

**Figure 5:**
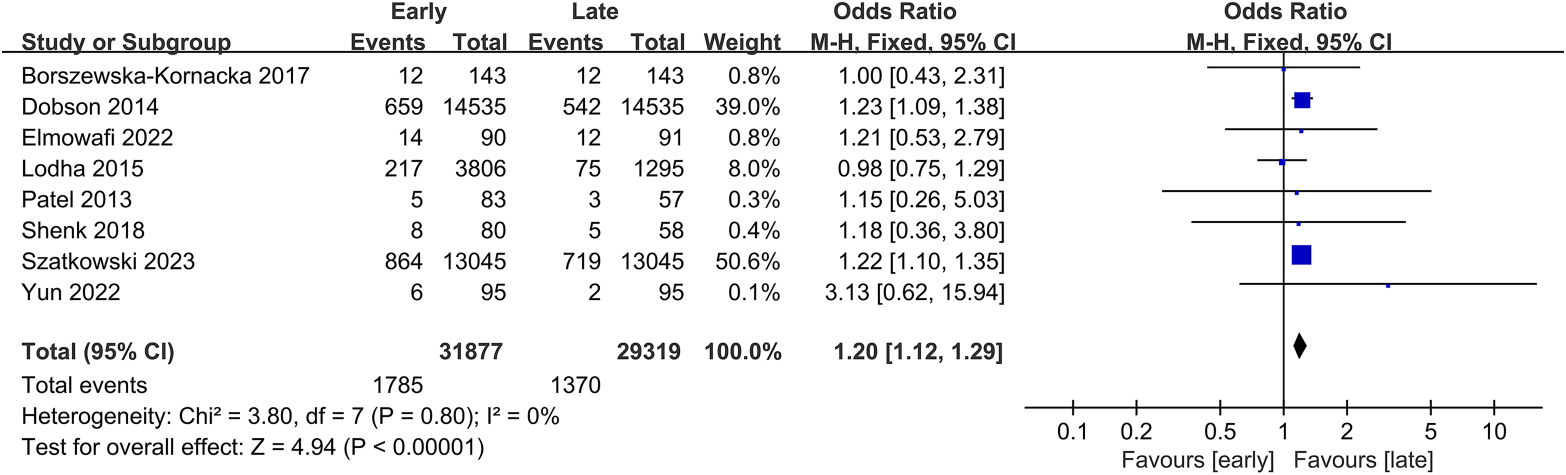
Forest plots of mortality.

Mortality data from 8 studies included 61,196 patients (31,877 in the early group vs. 29,319 in the late group) (10–14, 26, 27, 29). This study revealed a significantly higher mortality in the early group (OR: 1.20; 95% CI: 1.12, 1.29; *P* < 0.00001) with no significant heterogeneity (I^2^ = 0%, *P* = 0.80) (Figure 5).

Subgroup analysis showed a significant difference in both retrospective studies (OR: 1.19; 95% CI: 1.08, 1.31; *P* = 0.0004) (11–13, 29) and prospective studies (OR: 1.22; 95% CI: 1.09, 1.37; *P* = 0.0006) (Supplementary Figure S3) (10, 14, 26).

### 3.6 Sensitivity analysis and publication bias

Sensitivity analyses were conducted to assess the impact of individual studies on the pooled odds ratio (OR) for BPD incidence and mortality, with each eligible study sequentially excluded. The analyses demonstrated consistent and robust ORs following the exclusion of each study, both for BPD incidence (Supplementary Figure S4a) and mortality (Supplementary Figure S4b). Additionally, funnel plots and Egger's regression tests for BPD incidence and mortality, revealing no significant publication bias (Egger's test *P* = 0.463 for BPD incidence, Supplementary Figure S5a; *P* = 0.898 for mortality, Supplementary Figure S5b).

## 4 Discussion

Studies have confirmed that BPD is a combination of high oxygen exposure, barometric volume injury and inflammatory factors that lead to impaired lung maturity and abnormal development under the premise of genetic susceptibility, which seriously affects the quality of life of patients in the later period and increases the family economic burden (30, 31). At present, mechanical ventilation and inflammatory response are considered to be the main pathogenic mechanisms of BPD (30). First of all, most children with BPD received respiratory support after birth, which may bring a certain degree of lung damage. Premature lung tissue development was incomplete, which was easy to cause abnormal lung repair and lead to BPD (30). Secondly, inflammatory factors were considered to be the main cause of BPD, and the main pathogenic mechanism was currently believed to be the damage and repair imbalance caused by lung inflammation (32, 33). Studies have shown that inflammation inhibits the secretion of fibroblast growth factor-10 by type I alveolar epithelial cells through the NF-κB pathway, hindering secondary pulmonary segregation and leading to alveolar simplification (34). Therefore, the main factor to prevent the onset of BPD was to withdraw respiratory support as soon as possible to reduce and prevent the occurrence of inflammatory response (31).

Here, the efficacy of early and late caffeine therapy for BPD in infants were assessed. Our study revealed that early administration of caffeine can significantly mitigate the risk of BPD in preterm infants. Caffeine functions as a non-specific antagonist for adenosine receptors, which are present in four forms (A1, A2a, A2b, and A3) throughout the human body (35). Adenosine regulates the concentration of adenylate cyclase by binding to receptors in the body, and is involved in physiological processes such as inflammation, traumatic stress, ischemia reperfusion injury, and vascular dilation, while caffeine mainly acts on the first two receptors (36). Current studies believed that caffeine mainly prevents BPD through the following aspects. First of all, the high concentration of continuous oxygen exposure and the chemotaxis of inflammatory factors after delivery jointly acted on the lungs of newborns, increasing the lung fatigue of newborns and improving their respiratory resistance. Caffeine could improve the outcome of lung development by increasing the sensitivity of respiratory center to carbon dioxide, enhancing respiratory drive, improving lung ventilation, regulating lung vascular development, and inhibiting lung remodeling (37). Secondly, numerous studies have demonstrated that prolonged exposure to high concentrations of oxygen induces neutrophil infiltration into lung tissue and triggers production of inflammatory factors such as neutrophil chemokine-1, tumor necrosis factor-alpha (TNF-α), and interleukin-6 (IL-6). These factors adversely affect alveolar cell development and disrupt pulmonary capillary growth. Caffeine mitigates lung damage by inhibiting the entry of inflammatory factors into lung tissue.

However, we conducted subgroup analysis for the incidence of BPD by study type and found that the significant effect of early application of caffeine in reducing the incidence of BPD disappeared in the prospective study but remained in the retrospective study. Considering that prospective studies (including cohort studies and RCTs) control for the risk of confounders and bias better than retrospective studies, this difference urges caution in interpreting the association between early caffeine use and BPD incidence. Larger, multicenter, well-designed prospective studies are needed to confirm our findings.

On the other hand, infants with early caffeine use had a higher mortality rate. However, this result was somewhat influenced by the two largest cohort studies, both of which reported higher mortality in the early caffeine use group (10, 13), while other studies did not find a significant difference in mortality. Although this finding raises concerns about early caffeine use, higher mortality existed in early caffeine use group may be related to survival bias, as most infant deaths occur in the early years after birth (10). For example, infants who consume caffeine early (0–2 days after birth) are at risk of dying as early as 4 days after birth. In contrast, babies in the late caffeine group who received caffeine on the fifth day of life were apparently less likely to die on the fourth day of life. Further research is needed to confirm whether this conclusion is reliable.

There were several limitations. First of all, most included literatures were retrospective cohort studies, and the number of prospective studies was insufficient, especially RCTs with higher evidence quality, which is also one of the potential reasons leading to significant heterogeneity for the incidence of BPD. However, we did not observe a reduction in heterogeneity after subgroup analysis based on study type, so we speculate that it may be related to caffeine dose, patient sample size, study region and other factors. Secondly, the sample size of the literatures included in this meta-analysis is quite different, resulting in the final result being greatly affected by the large sample size of the studies. However, our sensitivity analysis results did not find instability. Moreover, most literature only reports the relationship between the timing of caffeine use and the mortality and incidence of BPD in the early postnatal period, and its long-term effect lacks effective data, which needs further research to confirm. This meta-analysis presents the latest evidence-based medical evidence and potential confounders for early caffeine use to reduce the incidence of BPD, as well as a potential positive association between early caffeine use and infant mortality, providing a theoretical basis for further prospective clinical studies. More large-scale, multi-center, prospective studies are needed to further confirm our findings.

## Data Availability

The original contributions presented in the study are included in the article/Supplementary Material, further inquiries can be directed to the corresponding author.
