# Supplementary material for: Early caffeine therapy decreases bronchopulmonary dysplasia but might increase mortality in preterm infants? a systematic review and meta-analysis
Source: Front Pediatr. 2025 Feb 21;13:1528054. doi: 10.3389/fped.2025.1528054 (PMC11885309; doi:10.3389/fped.2025.1528054)
Supplement: Supplementary file 2 [file Datasheet2.docx]

**Supplementary materials**

TableS1 The search strategy

| **Database** | **Search strategy** | **Number** |
| --- | --- | --- |
| Pubmed | (((("Caffeine"[Mesh]) OR ((((((((((((No Doz) OR (Caffedrine)) OR (Coffeinum N)) OR (Coffeinum Purrum)) OR (Dexitac)) OR (Durvitan)) OR (Percoffedrinol N)) OR (Vivarin)) OR (Percutaféine)) OR (Quick-Pep)) OR (QuickPep)) OR (Quick Pep))) OR ("methylxanthine" [Supplementary Concept])) OR ("Xanthine"[Mesh])) AND (("Bronchopulmonary Dysplasia"[Mesh]) OR (BPD)) | 180 |
| Embase | ((Caffeine or (No Doz or Caffedrine or Coffeinum N or Coffeinum Purrum or Dexitac or Durvitan or Percoffedrinol N or Vivarin or Percutafeine or Quick-Pep or QuickPep or Quick Pep) or methylxanthine or Xanthine) and (Bronchopulmonary Dysplasia or BPD)).af. | 551 |
| Cochrane | ((Caffeine or (No Doz or Caffedrine or Coffeinum N or Coffeinum Purrum or Dexitac or Durvitan or Percoffedrinol N or Vivarin or Percutafeine or Quick-Pep or QuickPep or Quick Pep) or methylxanthine or Xanthine) and (Bronchopulmonary Dysplasia or BPD)).af. | 78 |
| Web of Science | ((((Caffeine) OR ((((((((((((No Doz) OR (Caffedrine)) OR (Coffeinum N)) OR (Coffeinum Purrum)) OR (Dexitac)) OR (Durvitan)) OR (Percoffedrinol N)) OR (Vivarin)) OR (Percutafeine)) OR (Quick-Pep)) OR (QuickPep)) OR (Quick Pep))) OR (methylxanthine)) OR (Xanthine)) AND ((Bronchopulmonary Dysplasia) OR (BPD)) (All Fields) | 381 |

Table S2. Quality evaluation of the eligible studies with Newcastle–Ottawa scale.

| Study | Selection | | | | Comparability | | Outcome | | |
| --- | --- | --- | --- | --- | --- | --- | --- | --- | --- |
|  | Representative-ness | Selection of  non-exposed | Ascertainment  of exposure | Outcome not present at start | Comparability on most important factors | Comparability on other risk factors | Assessment of outcome | Long enough follow-up (median≥3 months) | Adequacy  (completeness) of follow-up |
| Borszewska-Kornacka 2017 | * | * | * | * | * | * | * | - | * |
| Dobson 2014 | * | * | * | * | * | - | * | - | * |
| Hand 2016 | * | * | * | * | * | * | * | - | * |
| Lodha 2015 | * | * | * | * | - | - | * | - | * |
| Patel 2013 | * | * | * | * | - | * | * | - | * |
| Shenk 2018 | * | * | * | * | - | * | * | - | * |
| Szatkowski 2023 | * | * | * | * | - | - | * | - | * |
| Taha 2014 | * | * | * | * | - | - | * | - | * |
| Ye 2023 | * | * | * | * | - | - | * | - | * |
| Yun 2022 | * | * | * | * | - | - | * | - | * |
| *indicates criterion met; - indicates significant of criterion not met. | | | | | | | | | |


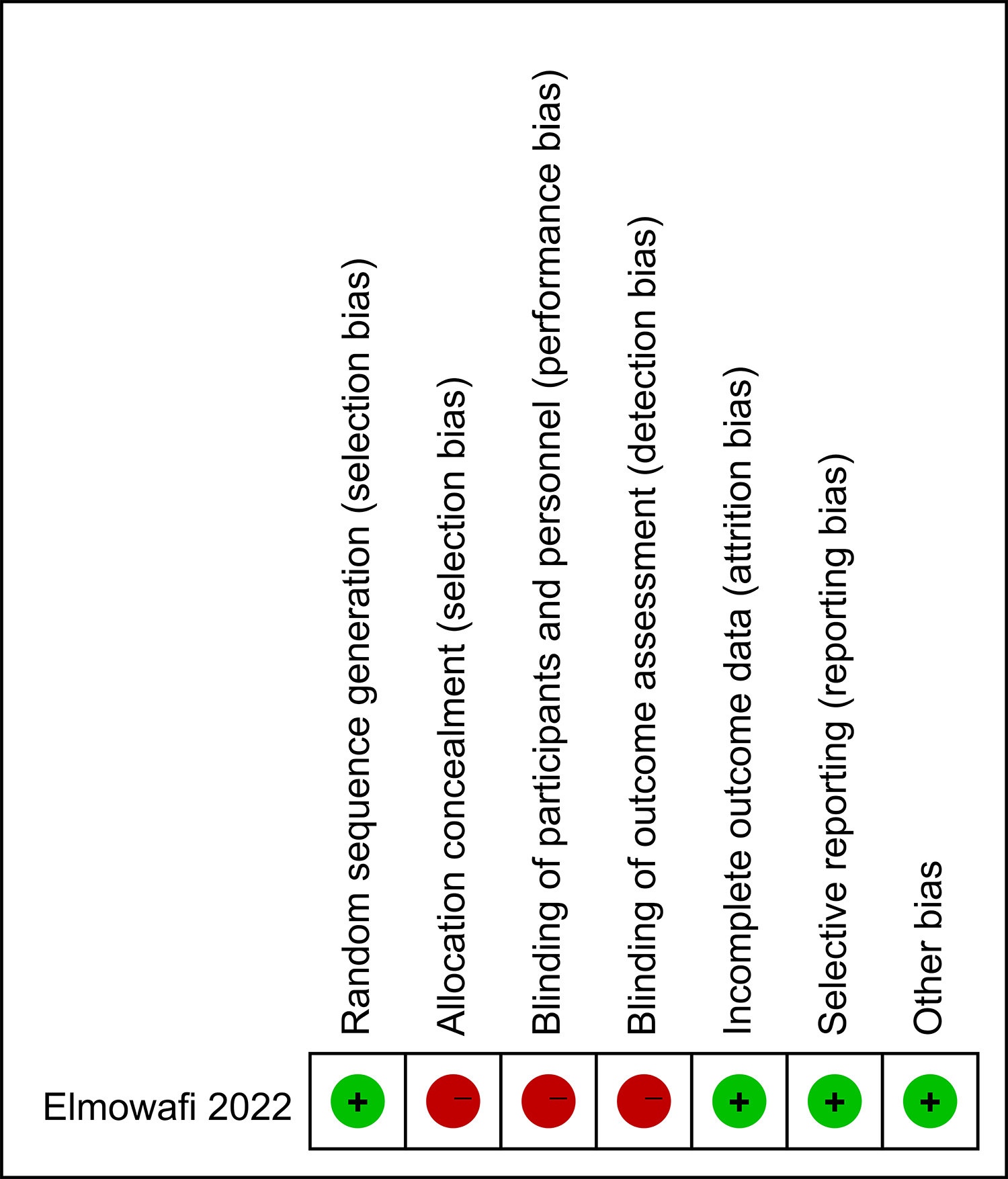


**Figure S1** Details of the quality evaluation for included RCT


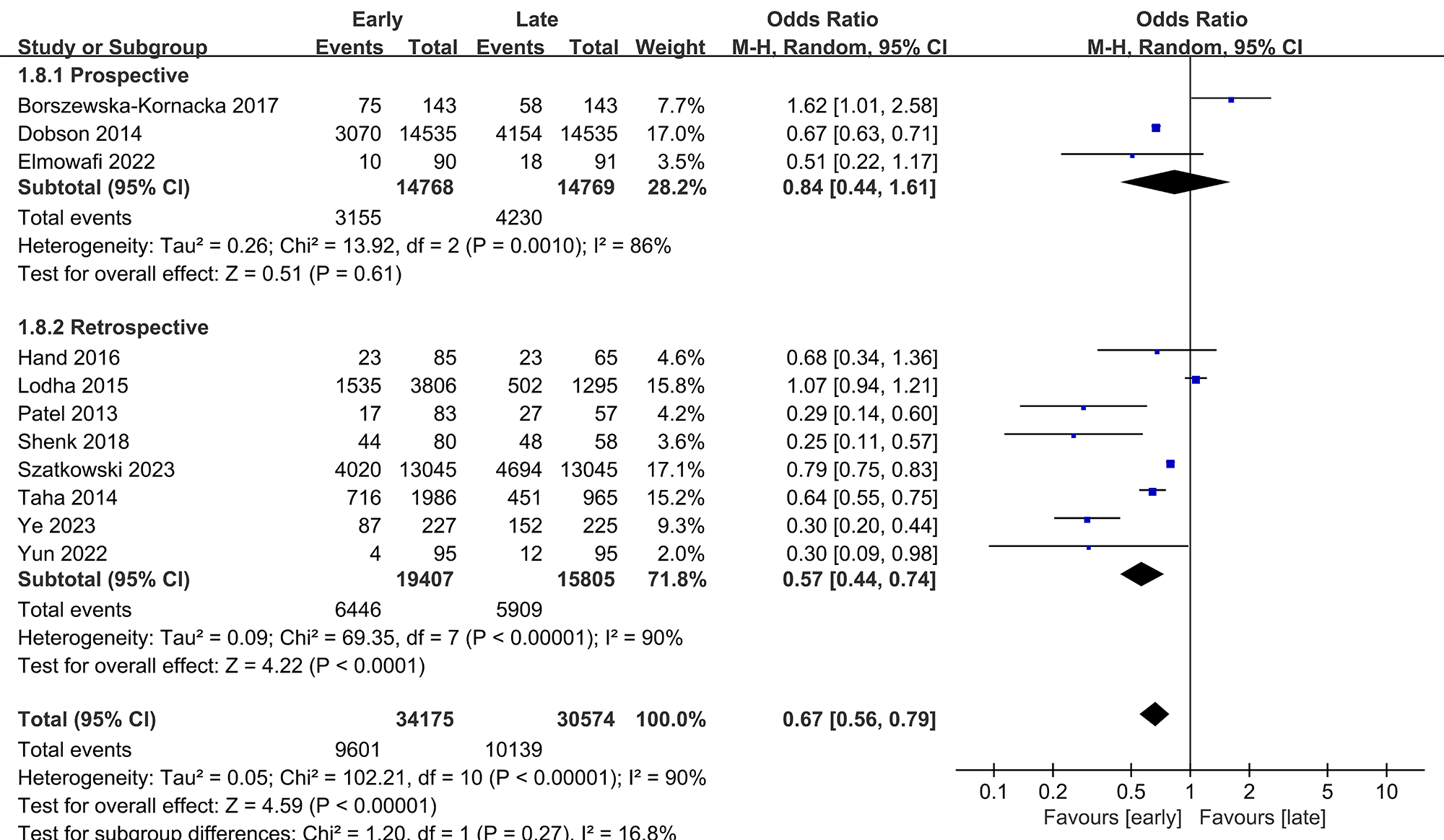


**Figure S2** Forest plots of subgroup analysis of the incidence of BPD


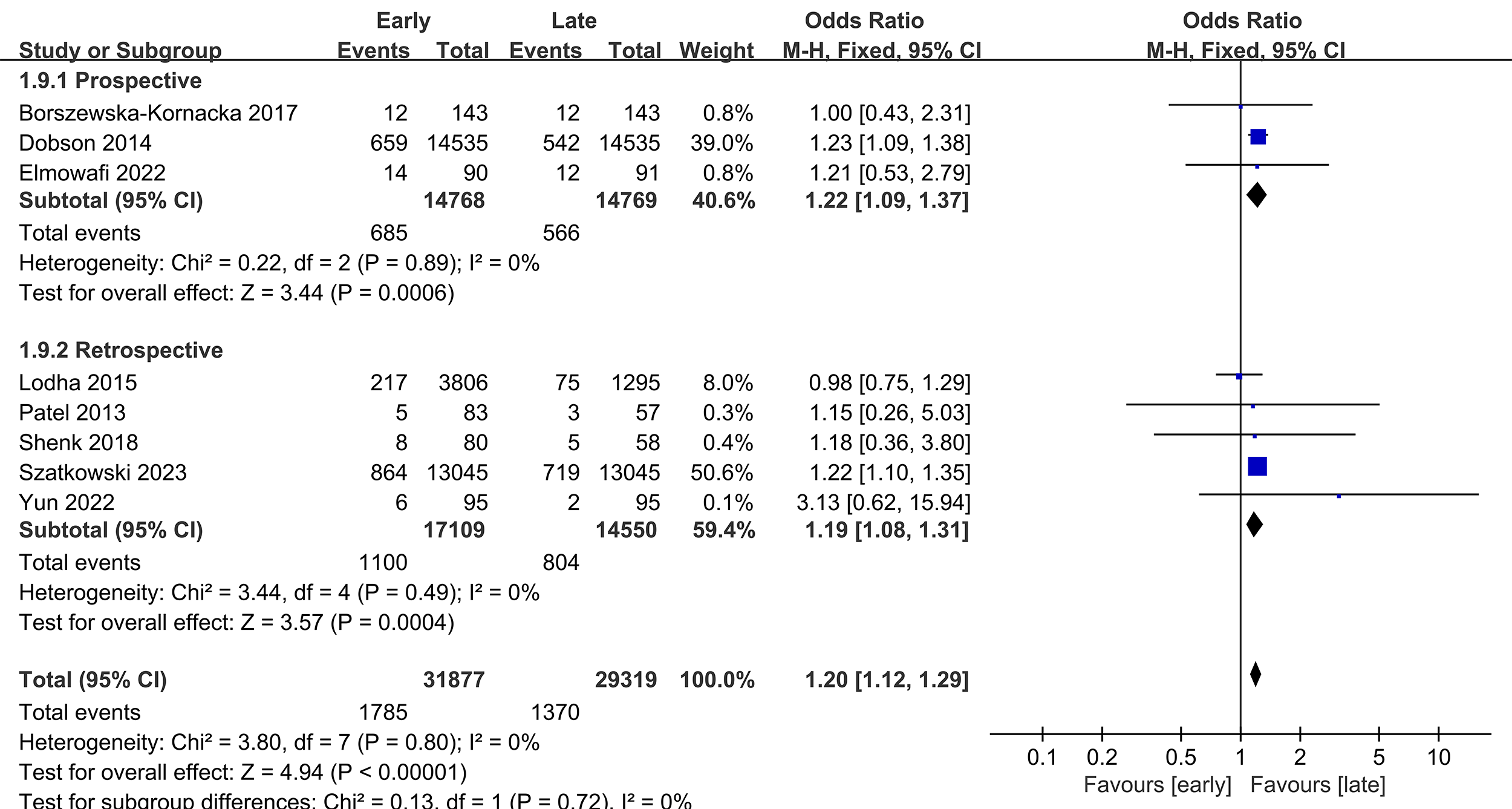


**Figure S3** Forest plots of subgroup analysis of mortality


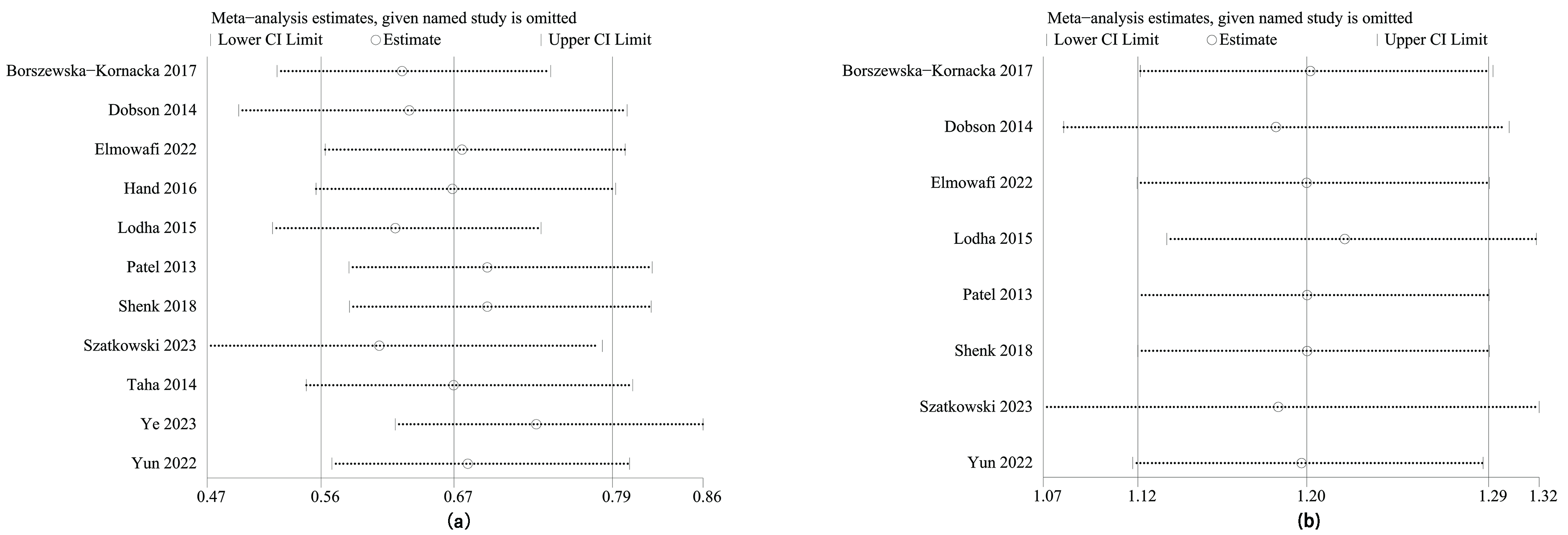


**Figure S4** Sensitivity analysis of (a) the incidence of BPD and (b) mortality


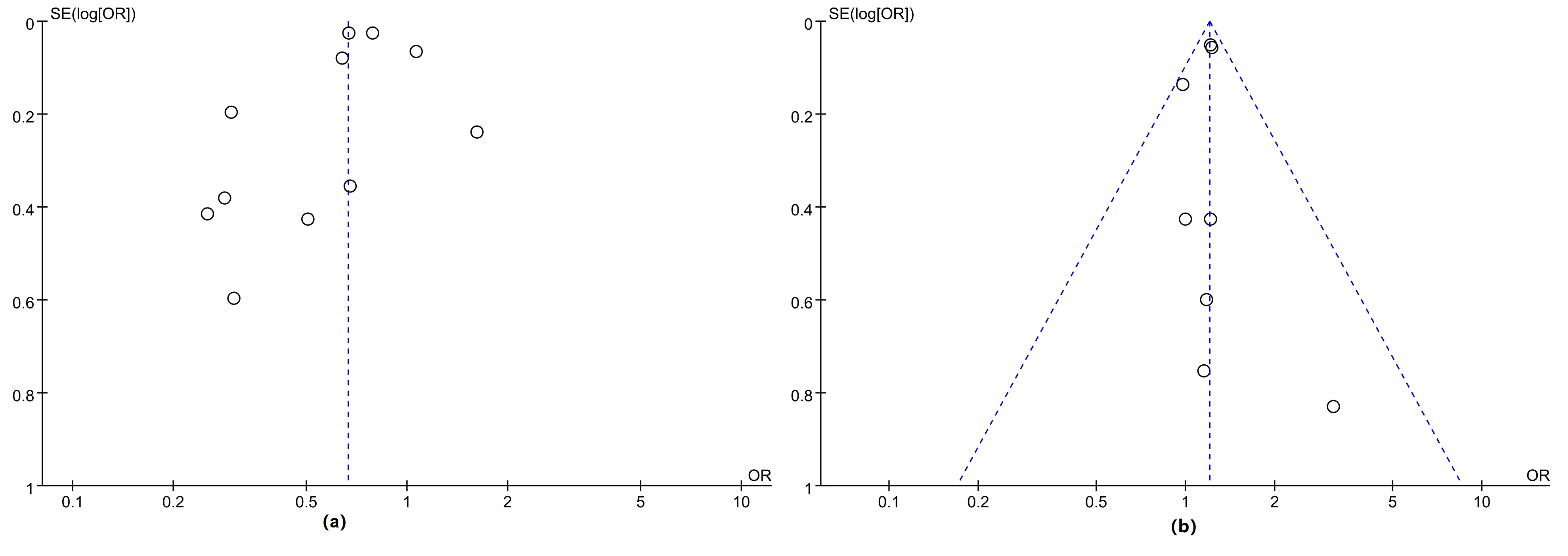


**Figure S5** Funnel plots of (a) the incidence of BPD and (b) mortality
